# Seizing opportunities for intervention: Changing HIV-related knowledge among men who have sex with men and transgender women attending trusted community centers in Nigeria

**DOI:** 10.1371/journal.pone.0229533

**Published:** 2020-03-02

**Authors:** Milissa U. Jones, Habib O. Ramadhani, Sylvia Adebajo, Charlotte A. Gaydos, Afoke Kokogho, Stefan D. Baral, Rebecca G. Nowak, Julie A. Ake, Hongjie Liu, Manhattan E. Charurat, Merlin L. Robb, Trevor A. Crowell, for the TRUST/RV368 Study Group

**Affiliations:** 1 Uniformed Services University, Bethesda, Maryland, United States of America; 2 Institute of Human Virology, University of Maryland School of Medicine, Baltimore, Maryland, United States of America; 3 Population Council Nigeria, Abuja, Nigeria; 4 Johns Hopkins University School of Medicine, Baltimore, Maryland, United States of America; 5 U.S. Army Medical Research Directorate – Africa/Nigeria, Abuja, Nigeria; 6 HJF Medical Research International, Abuja, Nigeria; 7 Johns Hopkins Bloomberg School of Public Health, Baltimore, Maryland, United States of America; 8 U.S. Military HIV Research Program, Walter Reed Army Institute of Research, Silver Spring, Maryland, United States of America; 9 University of Maryland School of Public Health, College Park, Maryland, United States of America; 10 Henry M. Jackson Foundation for the Advancement of Military Medicine, Bethesda, Maryland, United States of America; Ohio State University, UNITED STATES

## Abstract

**Background:**

Knowledge of HIV risk factors and reduction strategies is essential for prevention in key populations such as men who have sex with men (MSM) and transgender women (TGW). We evaluated factors associated with HIV-related knowledge among MSM and TGW and the impact of engagement in care at trusted community health centers in Nigeria.

**Methods:**

The TRUST/RV368 cohort recruited MSM and TGW in Lagos and Abuja, Nigeria via respondent driven sampling. During study visits every three months, participants underwent structured interviews to collect behavioral data, received HIV education, and were provided free condoms and condom compatible lubricants. Five HIV-related knowledge questions were asked at enrollment and repeated after 9 and 15 months. The mean number of correct responses was calculated for each visit with 95% confidence intervals (CIs). Multivariable Poisson regression was used to calculate adjusted risk ratios and CIs for factors associated with answering more knowledge questions correctly.

**Results:**

From March 2013 to April 2018, 2122 persons assigned male sex at birth were enrolled, including 234 TGW (11.2%). The mean number of correct responses at enrollment was 2.36 (95% CI: 2.31–2.41) and increased to 2.95 (95% CI: 2.86–3.04) and 3.06 (95% CI: 2.97–3.16) after 9 and 15 months in the study, respectively. Among 534 participants who completed all three HIV-related knowledge assessments, mean number of correct responses rose from 2.70 (95% CI: 2.60–2.80) to 3.02 (95% CI: 2.93–3.13) and then 3.06 (95% CI: 2.96–3.16). Factors associated with increased overall HIV-related knowledge included longer duration of study participation, HIV seropositivity, higher education level, and more frequent internet use.

**Conclusions:**

There was suboptimal HIV-related knowledge among Nigerian MSM and TGW at that improved modestly with engagement in care. These data demonstrate unmet HIV education needs among Nigerian MSM and TGW and provide insights into modalities that could be used to address these needs.

## Introduction

Globally, men who have sex with men (MSM) and transgender women (TGW) bear a disproportionate burden of HIV as compared to the general population [1–3]. In Nigeria, like many other parts of the world where same-sex sexual practices are criminalized, issues of discrimination, stigma, and fear create barriers to care and impede implementation of HIV preventive strategies[4–9], contributing to a disproportionate burden of HIV and other sexually transmitted infections[10–12]. The 2017 UNAIDS Databook noted an HIV prevalence of 23% among MSM in Nigeria and we have previously reported HIV prevalence as high as 66% among MSM and TGW in Lagos [13, 14]. MSM account for 10% of all new HIV infections in the country [15]. At present, epidemiologic reporting on HIV among transgender persons is scarce in Nigeria.

Establishing a foundation of accurate knowledge about HIV and its transmission, particularly among key populations such as MSM and TGW, is a fundamental stepping stone to behavioral change and uptake of prevention interventions [16, 17]. Data regarding HIV knowledge among MSM and TGW in all regions of Africa remain under reported and the limited data that are available suggest that substantial knowledge gaps exist [6, 18–21]. Nigeria was one of only two countries that reported HIV knowledge data for MSM during the 2008 United Nations General Assembly Special Session (UNGASS), noting that only 44% of MSM could correctly identify preventive strategies for HIV transmission and also correctly reject major misconceptions. Specific HIV knowledge data among TGW in Africa are lacking. Among MSM in Africa, suboptimal knowledge of HIV has been associated with unprotected intercourse, lack of HIV testing and increased HIV prevalence [22–24].

Population-level measures of HIV knowledge can also serve as critical indicators HIV prevention service coverage and effectiveness. Understanding gaps in HIV knowledge among MSM and TGW living in Nigeria may inform resource allocation and adjustment of HIV prevention interventions to address these gaps. Our study evaluated changes in HIV knowledge after engagement in care at MSM- and TGW-focused community health centers and described factors associated with improved HIV knowledge among MSM and TGW in Abuja and Lagos, Nigeria.

## Materials and methods

### Study design and population

MSM and TGW participants were recruited for the TRUST/RV368 cohort using respondent-driven sampling (RDS) in Abuja and Lagos, Nigeria, as previously described [7, 20, 25]. Eligibility required adulthood (age ≥16 years in Abuja; ≥18 years in Lagos), assignment of male sex at birth, one or more self-reported encounters of receptive or inserted anal intercourse within the previous 12 months, and a valid RDS coupon.

Upon enrollment and every three months thereafter, participants underwent a structured interview to collect demographic and behavioral data as well as a comprehensive medical examination by a study physician. Each visit included testing for HIV and other sexually transmitted infections with treatment provided as necessary, including antiretroviral therapy for HIV. Community centers were intended to convey a secure environment and build trust with participants through highly trained, engaged and compassionate study staff. Participants received individualized counseling about preventing HIV acquisition and transmission provided by study staff that were specially-trained in MSM and TGW healthcare needs. As previously described, counseling included education regarding safer sex practices specifically related to the benefit and proper use of condoms and condom compatible lubricants [26]. Additionally, throughout clinical areas and non-clinical community spaces, both condoms and water-based lubricants were made widely accessible, at no cost, to all participants.

Participants who enrolled in the cohort between March 2013 and April 2018, with documented responses to the HIV-related knowledge questions at the time of enrollment, were included in these analyses.

### Assessment of HIV-related knowledge

Participant knowledge of HIV prevention and transmission was assessed during the structured interview via five multiple-choice questions: *What type of sex puts you at risk for HIV infection*? *Which type of anal sex position puts you most at risk for HIV infection*? *Which is the safest lubricant to use during vaginal sex with a woman with latex condoms*? *Which is the safest lubricant to use during anal sex with latex condoms*? *Can you get HIV from using a needle to inject a drug or substance after someone*? The response choices are listed in (S1 Fig). These five questions were asked at enrollment and again after 9 and 15 months in care.

### Ethics

Written, voluntary, informed consent was provided by all study participants prior to enrollment. The study protocol was approved by institutional review boards at the Nigerian Federal Capital Territory and Nigerian Ministry of Defense, Abuja, Nigeria; the University of Maryland, Baltimore, MD, USA; and the Walter Reed Army Institute of Research, Silver Spring, MD, USA.

### Data capture and analysis

The response to each question was documented on paper case report forms and imported into the research database using TeleForm (Hewlett-Packard Inc., Palo Alto, CA, USA) data capture system. Trained personnel confirmed the accuracy of each data capture.

The population-averaged mean number of correct responses to HIV knowledge questions was calculated at enrollment, 9, and 15 months in the study. Exact 95% confidence intervals (CIs) were calculated assuming a normal distribution. Multivariable Poisson regression with generalized estimating equations was used to calculate risk ratios (RRs) and 95% CIs for factors associated with answering more knowledge questions correctly. Pearson’s chi-squared test was used to compare the proportion of participants answering each question correctly at enrollment and subsequent visits. In sensitivity analyses, the study population was restricted to participants with HIV knowledge assessments at all three visits of interest in order to remove the impact of differential loss to follow-up in the study. All analyses were performed using Stata 15.0 (StataCorp LP, College Station, TX, USA).

## Results

### Study population

From March 2013 to April 2018, 2122 people who were assigned male gender at birth were enrolled in the TRUST/RV368 cohort and completed the enrollment questionnaire assessing HIV knowledge. In general, participants were young (median age 23, interquartile range 21–27) and the majority self-identified as men (80.0%) and bisexual (65.6%) (Table 1). There were 234 (11.0%) transgender women. Among 1,709 participants with HIV testing results, 851 (49.8%) were seropositive.

**Table 1:** Demographic and behavioral characteristics of a cohort of Nigerian MSM and TGW who completed HIV knowledge assessment(s) from 2013–2018.

Characteristic	N = 2122 (%)
**Age**	
Median (IQR)	23 (21–27)
≤ 21 years	701 (33.0)
22–30 years	1201 (56.6)
>30 years	220 (10.4)
**Gender Identity**	
Man	1697 (80.0)
Woman	234 (11.0)
Other/Unknown	191 (9.0)
**Sexual Orientation**	
Gay/Homosexual	715 (33.7)
Bisexual	1392 (65.6)
Other/Unknown	15 (0.7)
**Religion**	
Christian	1476 (69.6)
Muslim	628 (29.6)
None/Other/Unknown	18 (0.8)
**Education level**	
Junior Secondary or Less	309 (14.6)
Senior Secondary	1108 (52.2)
Higher than Senior Secondary	693 (32.7)
Unknown	12 (0.6)
**Occupation**	
Unemployed	342 (16.1)
Student	400 (18.9)
Professional/Self-Employed	403 (19.0)
Entertainment/Hospitality	215 (10.1)
Driver/Laborer	43 (2.0)
Other/Unknown	719 (33.9)
**Marital Status**	
Single/Never Married	1880 (88.6)
Married/Living with a Woman	145 (6.8)
Living with a Man	29 (1.4)
Divorced/Separated/Widowed/Other	68 (3.2)
**Own a Mobile Phone**	
No	146 (6.9)
Yes	1958 (92.3)
Unknown	18 (0.8)
**Internet Use**	
Never	487 (23.0)
Less than once a week	416 (19.6)
Almost everyday	1199 (56.5)
Other/Unknown	20 (0.9)
**HIV Status**	
Negative	858 (40.4)
Positive	851 (40.1)
Unknown	413 (19.5)
**Location**	
Abuja	1450 (68.3)
Lagos	672 (31.7)

IQR = Interquartile Range.

There were 534 participants who completed HIV-related knowledge assessments at both 9 and 15 months after enrollment, comprising the more restricted study population used for sensitivity analyses. This group differed from the general study population in several ways, including trends toward older age, higher education level, more frequent internet use, and greater likelihood of being HIV-infected (S1 Table). They were also substantially more likely to have been recruited from the Lagos study site.

### Responses to HIV knowledge questions and change over time

**Fig 1:**
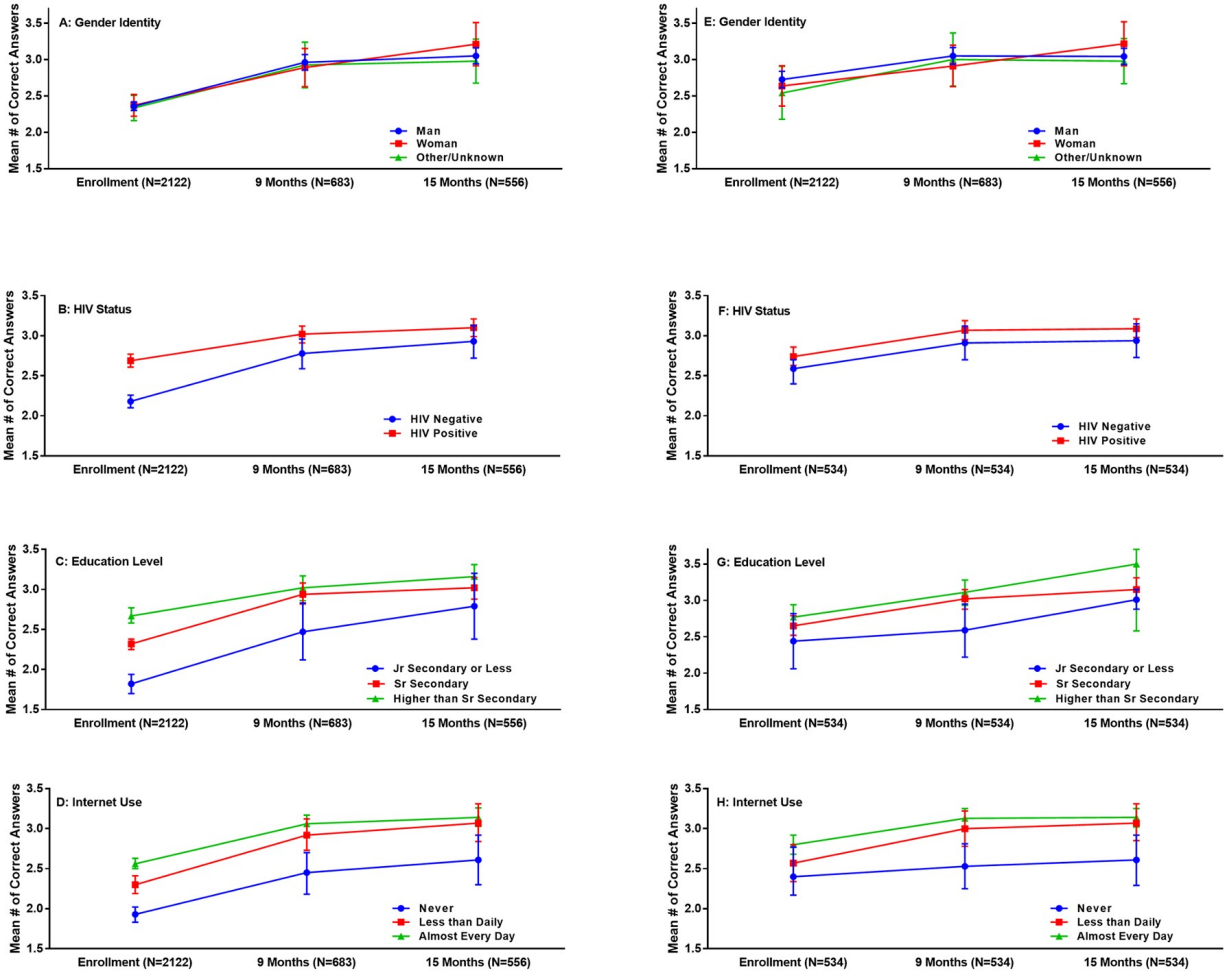
Mean number of correct responses to HIV knowledge questions among cohort of MSM and TGW in Nigeria from 2013–2018, by participant factors. **A-D**: Mean number of correct responses over time in cohort by participant factors (all participants); (A) Gender Identity (B) HIV Status (C) Education Level (D) Internet Use **E-H**: Mean number of correct response over time in cohort, by participant factors (participants completing all follow up visits) (E) Gender Identity (F)HIV Status (G)Education Level (H)Internet Use. Abbreviations: MSM: men who have sex with men; TGW: transgender women.

The mean number of correct responses to the five HIV knowledge questions at the time of enrollment was 2.36 (95% CI: 2.31–2.41). This rose to 2.95 (95% CI: 2.86–3.04) after 9 months and 3.06 (95% CI: 2.97–3.16) after 15 months in the study. When stratified by gender identity, there was no statistically significant differences observed (Fig 1A). Increases were observed in other subgroups when stratifying by HIV status, education level, and internet use (Fig 1B–1D). Among the 534 participants who completed all three longitudinal assessments of HIV knowledge, the mean number of correct responses rose from 2.70 (95% CI: 2.60–2.80) to 3.02 (95% CI: 2.93–3.13) and then 3.06 (95% CI: 2.96–3.16) at enrollment, 9 and 15 months, respectively. Again, when stratified by gender identity among this subpopulation, there was no statistically significant differences observed (Fig 1E). Increases were observed in other subgroups when stratifying by HIV status, education level, and internet use among this subgroup as well (Fig 1F–1H).

**Fig 2:**
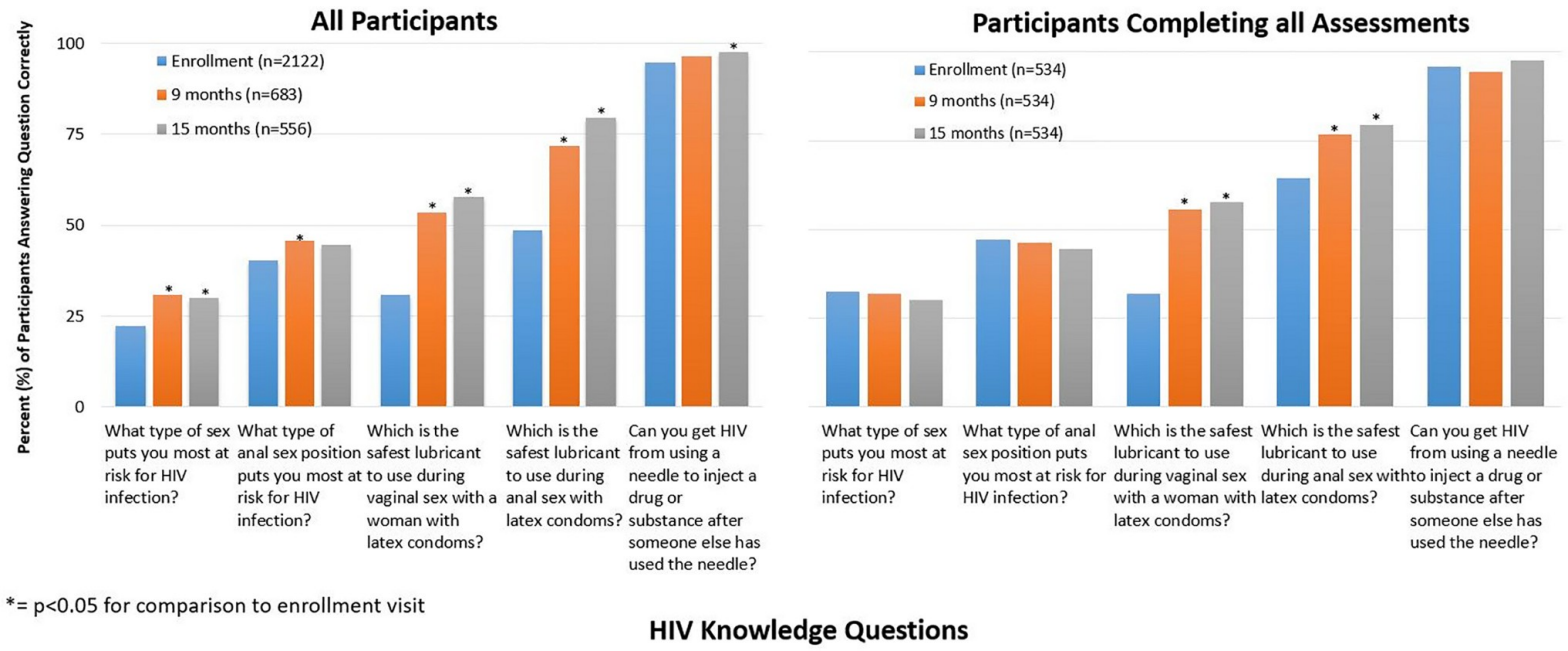
Percentage of correct responses to individual HIV knowledge questions among cohort of MSM and TGW in Nigeria from 2013–2018. Abbreviations: MSM: men who have sex with men; TGW: transgender women (TGW). * = p<0.05 for comparison to enrollment visit.

At the time of enrollment, only 21.7% of participants correctly identified anal sex when asked, ‘*What type of sex puts one at most risk for HIV transmission*?*’* By the 15-month follow-up visit, the percentage of participants responding correctly increased to 29.5% (p<0.001; Fig 2A). At enrollment, 40.4% of participants correctly reported that receptive anal sex puts one at higher risk for HIV transmission as compared to insertive anal sex and this remained steady at 43.5% at the 15-month follow-up visit (p = 0.180). The percentages of participants who correctly identified water-based lubricants as the safest for use with latex condoms increased when considering both vaginal (from 30.7% to 57.4%. p<0.001) and anal (48.3% to 78.8%, p<0.001) sex. Almost all participants were aware of the risk of HIV from intravenous drug use at the time of enrollment (94.8%) and at the 15-month visit (97.3%, p = 0.012).

Among participants who completed all three longitudinal assessments of HIV knowledge, only the percentages of participants correctly identifying the safest lubricant to use with latex condoms during vaginal and anal intercourse increased over time (Fig 2B). In contrast to the primary analysis, in the more restricted population there was no increase in the number of participants correctly identifying the type of sex associated with greatest risk of HIV or that HIV transmission could occur via needle sharing.

### Factors associated with increased HIV knowledge

Unadjusted and adjusted analyses of factors associated with increased HIV knowledge are presented in Table 2. Increased duration of study participation was independently associated with increased HIV knowledge. HIV seropositivity as well as increased education level were also associated with increased HIV knowledge. Lastly, internet usage of less than daily or almost every day was associated with increased HIV knowledge in comparison to participants who never used the internet.

**Table 2:** Characteristics of a cohort of Nigerian MSM and TGW associated with increasing number of correct responses to HIV knowledge questions.

Characteristic	Unadjusted Risk Ratio (95% CI)	Adjusted Risk Ratio (95% CI)
**Visit Number**		
Enrollment	Reference	
9 Months	**1.21 (1.15–1.27)**	**1.15 (1.04–1.27)**
15 Months	**1.25 (1.19–1.32)**	**1.19 (1.07–1.32)**
**Age**		
≤ 21 years	Reference	
22–30	**1.12 (1.06–1.18)**	1.04 (0.98–1.10)
>30	**1.12 (1.03–1.21)**	1.04 (0.95–1.14)
**Gender Identity**		
Male	Reference	
Female	1.00 (0.92–1.09)	0.99 (0.91–1.08)
Other/Unknown	**1.20 (1.15–1.25)**	0.98 (0.89–1.08)
**Sexual Orientation**		
Gay/Homosexual	Reference	
Bisexual	0.99 (0.96–1.05)	0.98 (0.91–1.06)
Other/Unknown	0.67 (0.48–0.94)	0.78 (0.40–1.51)
**Education Level**		
Junior Secondary or Less	Reference	Reference
Senior Secondary	**1.30 (1.20–1.42)**	**1.12 (1.02–1.23)**
Higher than Senior Secondary	**1.47 (1.35–1.61)**	**1.22 (1.10–1.34)**
Unknown	1.16 (0.83–1.63)	1.15 (0.82–1.60)
**Occupation**		
Unemployed	Reference	
Student	1.06 (0.98–1.15)	1.01 (.94–1.10)
Professional/Self-Employed	1.03 (0.95–1.12)	1.05 (0.97–1.14)
Entertainment/Hospitality	0.99 (0.90–1.08)	0.99 (0.90–1.08)
Driver/Laborer	1.05 (0.89–1.24)	1.06 (0.90–1.25)
Other/Unknown	1.05 (0.98–1.13)	0.98 (0.91–1.05)
**Marital Status**		
Single/Never Married	Reference	
Married/Living with a Woman	0.92 (0.83–1.02)	0.99 (.89–1.11)
Living with a Man	1.15 (0.96–1.38)	1.03 (.86–1.22)
Divorced/Separated/Widowed/Other	0.89 (0.78–1.03)	0.92 (0.80–1.06)
**Owns Mobile Phone**		
No	Reference	
Yes	**1.32 (1.17–1.48)**	1.05 (0.93–1.19)
Unknown	0.82 (0.58–1.15)	0.69 (0.47–1.02)
**Internet Use Frequency**		
Never	Reference	
Less than daily	**1.24 (1.14–1.34)**	**1.11 (1.02–1.21)**
Almost Daily	**1.36 (1.27–1.45)**	**1.18 (1.09–1.27)**
Unknown	0.97 (0.75–1.28)	1.11 (0.80–1.54)
**HIV Status**		
Negative	Reference	
Positive	**1.25 (1.19–1.31)**	**1.16 (1.10–1.22)**
Unknown	**0.90 (0.83–0.97)**	0.97 (0.90–1.06)
**Location**		
Abuja	Reference	Reference
Lagos	**1.16 (1.10–1.22)**	1.05 (1.00–1.11)

CI-Confidence Interval. Poisson regression with generalized estimating equations was used to calculate unadjusted and adjusted risk ratios (RRs) and 95% confidence intervals. The adjusted risk ratios reflect a multivariable model that included all listed variables. Statistically significant risk ratios (p<0.05) are in bold.

## Discussion

HIV knowledge improved over time in this study of MSM and TGW attending trusted community health centers in Abuja and Lagos, Nigeria. While improvements were noted across multiple domains when examining all study participants, the sensitivity analysis, limited to participants who completed all three HIV knowledge assessments, suggested that gains were primarily driven by improved knowledge about lubricant use during vaginal and anal sex. This outcome is important as prior research has shown that knowledge of the appropriate lubricant to use with latex condoms was associated with wearing condoms during the most recent sexual contact among MSM in Lesotho [27]. In sub-Saharan Africa, access to condom-compatible lubricants is impeded by both stigmatization and resource constraints [28, 29]. These factors contribute to frequent utilization of alternative sexual lubricants such as saliva, soap, butter, yogurt, and cooking oils [30]. In our study, water-based lubricants were freely available to study participants in conjunction with education about their use which may potentially account for the knowledge gains observed in this domain. The implementation of similar strategies at specialized facilities dedicated to MSM and TGW, coupled with periodic knowledge assessments, could result in sustained, measurable educational gains in the domain of appropriate lubricant use among this population.

It is important to note that although HIV knowledge improved over time in the study, HIV knowledge overall was suboptimal among our population which corroborates previously reported data regarding HIV knowledge among MSM in Africa [6, 18, 19, 21, 27]. During their time in study, participants were not routinely taught which sexual position puts one at greatest risk of HIV as a consensus regarding seropositioning as a mitigation tool in preventing HIV transmission had not yet been reached [31–33]. Another potential reason for suboptimal HIV knowledge over time in the study is the downstream effect of continued stigma and criminalization of HIV in Nigeria, where participants may have opted to limit the extent of their responses so as not to appear “too knowledgeable” about homosexual intercourse. Stakeholders in HIV prevention interventions need to identify the critical educational concepts to convey, tailor materials accordingly, and develop relevant knowledge assessment tools.

Certain participant characteristics that were associated with increasing HIV knowledge may highlight areas for potential intervention. Increased study engagement (completion of more than just the enrollment visit) was a factor independently associated with higher levels of HIV knowledge, which may indicate that consistent engagement with health care providers in facilities that are trusted by MSM and TGW may foster an environment in which HIV preventive messaging is solidified. HIV seropositivity was another factor associated with increased HIV knowledge, potentially reflecting the impact of education that occurs routinely as part of voluntary counseling and testing as well as subsequent HIV care. Participants completing higher than junior secondary education demonstrated increased HIV knowledge, which underscores the importance of access to education as a core intervention to target health behaviors and corroborates earlier research showing that higher education level was associated with greater knowledge of HIV and AIDS [18, 21, 34, 35].

Internet use was associated with increased HIV knowledge. Individuals with frequent internet use may be exposed to HIV educational materials, forums, and social networks, allowing a greater comprehension of HIV and associated risk and prevention strategies. Previous research in the United States and South Africa has shown that MSM who use the internet and social media can score highly on HIV knowledge assessments, but did not evaluate HIV knowledge among non-users of the internet [21]. Our finding that internet use is associated with improved knowledge suggests that internet-based educational interventions such as websites, online forums, and social media outlets may allow access to a larger breadth and depth of HIV preventive education in spaces in which shame, stigma and marginalization are minimized. A 2017 systematic review of 17 internet-based interventions that targeted HIV knowledge and risk behaviors among young MSM, demonstrated that all except one reported statistically significant changes in at least one primary outcome, indicating that such interventions showed promise to affect change among this population.[36].

We acknowledge strengths as well as limitations of our analyses. Employment of RDS allowed access to Nigerian MSM and TGW who may have otherwise been unreachable by traditional recruitment strategies. MSM and TGW are marginalized populations that are historically under-represented in studies from sub-Saharan Africa. Specific data related to HIV knowledge among TGW in Nigeria are scarce and our study adds substantially to the literature by demonstrating similar levels of HIV knowledge among TGW as compared to MSM in Nigeria. The use of standardized questionnaires via structured interviews allowed the ability to gather detailed information related to HIV knowledge and other participant characteristics. However, the HIV knowledge assessment questions utilized by our study were not standard HIV knowledge questions used by the Demographic and Health Surveys Program (DHS) thus may not be comparable to other HIV knowledge studies using this survey tool. Only about a quarter of participants who enrolled in the study were retained through month 15 and completed all three HIV knowledge assessments, introducing the possibility of biased results due to differential loss to follow-up. This group differed from the participants that completed less than three HIV knowledge assessments in key ways including: being more highly educated, more frequent users of the internet, and more likely to be living with HIV. However, most inferences were robust to sensitivity analyses that limited the study population to those with all three HIV knowledge assessments.

## Conclusions

HIV education is prerequisite to behavioral change and uptake of HIV prevention interventions. Our study demonstrated improved HIV knowledge during engagement in MSM- and TGW-focused counseling and care at trusted community centers in Nigeria, although substantial room for improvement persists. Access to, and education about, water-based lubricants accounted for the majority of the knowledge gains demonstrated in this population and similar strategies could be effectively deployed in other MSM- and TGW-focused clinical settings. Participants who remained engaged in care as well as those who utilized the internet frequently demonstrated increased HIV knowledge, suggesting that multiple education modalities could be leveraged to improve HIV knowledge and further reduce HIV transmission. Interventions centered on the provision of internet access and web-based delivery of HIV educational materials may be useful adjuncts to direct counseling at trusted health care centers in Nigeria and elsewhere.

## Supporting information

S1 Fig HIV-related knowledge questions and response choices asked during structured interviews in the TRUST/RV368 cohort study. (TIF)

S1 Table Sensitivity analysis comparing demographic and behavioral characteristics of Nigerian MSM and TGW that completed less than 3 HIV knowledge assessments compared to participants completing all HIV knowledge assessments. Abbreviations: MSM: men who have sex with men; TGW: transgender women; IQR = Interquartile Range.
(DOCX)
