# Supplementary figures and images for: Seizing opportunities for intervention: Changing HIV-related knowledge among men who have sex with men and transgender women attending trusted community centers in Nigeria
Source: PLoS One. 2020 Mar 2;15(3):e0229533. doi: 10.1371/journal.pone.0229533 (PMC7051043; doi:10.1371/journal.pone.0229533)

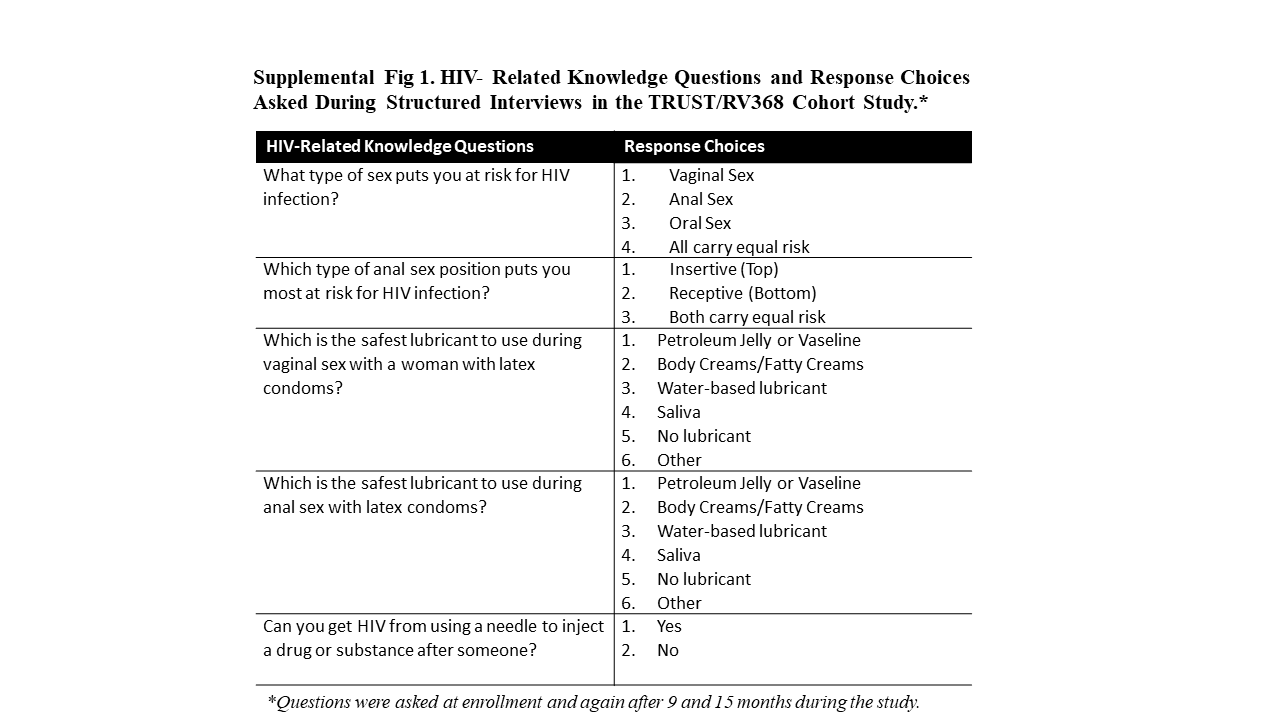

Supplement: S1 Fig — (TIF) [file pone.0229533.s001.tif]
