# Supplementary material for: Seizing opportunities for intervention: Changing HIV-related knowledge among men who have sex with men and transgender women attending trusted community centers in Nigeria
Source: PLoS One. 2020 Mar 2;15(3):e0229533. doi: 10.1371/journal.pone.0229533 (PMC7051043; doi:10.1371/journal.pone.0229533)
Supplement: S1 Table — Abbreviations: MSM: men who have sex with men; TGW: transgender women; IQR = Interquartile Range. (DOCX) [file pone.0229533.s002.docx]

| **Characteristic** | **< 3 HIV Knowledge Visits**  **N=1588 (%)** | **All HIV Knowledge Visits**  **N=534 (%)** | **p-Value** |
| --- | --- | --- | --- |
| **Age** |  |  |  |
| Median (IQR) | 23 (20-27) | 24 (21-27) | <0.001 |
| ≤ 21 Years | 558 (35.1) | 143 (26.8) |  |
| 22-30 Years | 879 (55.4) | 322 (60.3) |  |
| >30 Years | 151 (9.5) | 69 (12.9) |  |
| **Gender Identity** |  |  | 0.27 |
| Man | 1278 (80.5) | 419 (78.5) |  |
| Woman | 165 (10.4) | 69 (12.9) |  |
| Other/Unknown | 145 (9.1) | 46 (8.6) |  |
| **Sexual Orientation** |  |  |  |
| Gay/Homosexual | 515 (32.4) | 200 (37.5) | 0.10 |
| Bisexual | 1062 (66.9) | 330 (61.8) |  |
| Other/Unknown | 11 (0.7) | 4 (0.7) |  |
| **Religion** |  |  |  |
| Christian | 1048 (66.0) | 428 (80.1) | <0.001 |
| Muslim | 525 (33.1) | 103 (19.3) |  |
| None/Other/Unknown | 15 (0.9) | 3 (0.6) |  |
| **Education Level** |  |  |  |
| Junior Secondary or Less | 275 (17.3) | 34 (6.4) | <0.001 |
| Senior Secondary | 828 (52.1) | 280 (52.4) |  |
| Higher than Senior Secondary | 478 (30.1) | 216 (40.4) |  |
| Unknown | 7 (0.4) | 4 (0.7) |  |
| **Occupation** |  |  |  |
| Unemployed | 233 (14.7) | 109(20.4) | <0.001 |
| Student | 281 (17.7) | 119(22.3) |  |
| Professional/Self-Employed | 302 (19.0) | 101 (18.9) |  |
| Entertainment/Hospitality | 152 (9.6) | 63 (11.8) |  |
| Driver/Laborer | 29 (1.8) | 14 (2.6) |  |
| Other/Unknown | 591 (37.2) | 128 (24.0) |  |
| **Marital Status** |  |  |  |
| Single/Never Married | 1410 (88.8) | 470 (88.0) | <0.001 |
| Married/Living with A Woman | 120 (7.6) | 25 (4.7) |  |
| Living with A Man | 15 (0.9) | 14 (2.6) |  |
| Divorced/Separated/Widowed/Other | 43 (2.7) | 25 (4.7) |  |
| **Own A Mobile Phone** |  |  |  |
| No | 135 (8.5) | 11(2.1) | <0.001 |
| Yes | 1440 (90.7) | 518 (97.0) |  |
| Unknown | 13 (0.8) | 5 (0.9) |  |
| **Internet Use** |  |  |  |
| Never | 429 (27.0) | 58 (10.9) | <0.001 |
| Less Than Daily | 309 (19.5) | 107 (20.2) |  |
| Almost Everyday | 836 (52.6) | 363 (68.0) |  |
| Other/Unknown | 14 (0.9) | 6 (1.1) |  |
| **HIV Status** |  |  |  |
| Negative | 697 (43.9) | 161 (30.1) | <0.001 |
| Positive | 478 (30.1) | 373 (69.9) |  |
| Unknown | 413 (26.0) | 0 (0) |  |
| **Location** |  |  |  |
| Abuja | 1174 (73.9) | 276 (51.7) | <0.001 |
| Lagos | 414 (26.1) | 258 (48.3) |  |

**Supplemental Table 1. Sensitivity Analysis Comparing Demographic and Behavioral Characteristics of Nigerian MSM and TGW That Completed Less Than 3 HIV Knowledge Assessments Compared to Participants Completing all HIV Knowledge Assessments.**

Abbreviations: MSM: men who have sex with men; TGW: transgender women; IQR= Interquartile Range
